# Assessment of the prevalence of intestinal parasitosis and associated risk factors among primary school children in Chencha town, Southern Ethiopia

**DOI:** 10.1186/1471-2458-14-166

**Published:** 2014-02-14

**Authors:** Ashenafi Abossie, Mohammed Seid

**Affiliations:** 1 Medical Laboratory Technology Team, Arbaminch College of Health Sciences, P. O. Box 155, Arbaminch, Ethiopia

**Keywords:** Intestinal parasitic infections, Chencha, Risk factors, Soil transmitted helminthes

## Abstract

**Background:**

Parasitic infection is the most prevalent among rural communities in warm and humid regions and where water, hygiene and sanitation facilities are inadequate. Such infection occurs in rural areas where water supplies are not enough to drink and use, and in the absence of environmental sanitation, when the rubbish and other wastes increased, and sewage and waste water are not properly treated. Hence the aim of the study was to assess the prevalence of intestinal parasitosis and associated risk factors.

**Methods:**

This cross sectional study was conducted on children of the selected primary schools in Chencha town from March to May, 2012. Children were selected within age group 5–15 years. The socio-demographic, environmental and behavioral variables data were collected using structured questionnaire from the guardians of children and school teachers to assess the risk factors. Prevalence of intestinal parasitosis was determined using direct method and formol-ether concentration method. Participants’ data were analyzed using SPSS version 16.0.

**Results:**

Of 422 selected school children, 400 participated in the study with full information for analysis. The overall prevalence of intestinal parasitosis was high (81.0%). Soil-transmitted helminths (STHs) infections (63.0%) were more prevalent than protozoa infections (23.5%). The predominant parasites were *A.lumbricoides* (60.5%), *E.histolytica/dispar (*16.25%), *Giardia lamblia (*11.7%) and *T.trichuria* (9.7%). The presence of Intestinal Parasitic Infections (IPIs) have statistically significant association with the educational status of the household heads, absence of washing facility, home cleanness condition and type of latrine used with (p < 0.05).

**Conclusions:**

The prevalence of intestinal parasitic infections, especially soil-transmitted helminths (STHs) is very high in the school children. The high prevalence of parasitic infections in these children indicates that the protozoa and helminths concerned are very common in the environment of these villages and the results of the risk factors analysis suggests that the transmission is from several routes. Therefore, multiple intervention strategies should be implemented for the school children, households and the environment to reduce the disease burden.

## Background

Intestinal parasitic infections (IPIs) have been described as the greatest worldwide cause of illnesses and diseases [1]. Soil-Transmitted Helminths (STHs) together with parasitic intestinal protozoa are causes for a vast amount of morbidity, discomfort and often mortality in tropical and sub-tropical regions around the world [2]. People of all ages are affected by this cycle of prevalent parasitic infections; however, children are the most affected [3].

School children aged 5–15 years suffer the highest infection rate and worm burden that attributes to poor sanitation and hygiene [4]. About 400 million school-age children are infected with roundworm, whipworm and hookworm worldwide, a large proportion of whom are found in the East Asia region [5-7]. Distributions of IPIs are linked to lack of sanitation, lack of access to safe water and improper hygiene; therefore, they occur wherever there is poverty. IPIs deprive the poorest of the poor of health, contributing to economic instability and social marginalization.

Intestinal parasites, which have direct life cycle, are transmitted by faecal oral route to human through poor personal hygiene [2]. Apart from causing mortality and morbidity, infection with intestinal parasites has been associated with stunting of linear growth, physical weakness and low education achievement in school children [8].

These parasites consume nutrients from children they infect, thus retarding their physical development. They destroy tissues and organs, cause abdominal pain, diarrhea, intestinal obstruction, anemia, ulcers and other health problems which can lead to slow cognitive development and impaired learning [9].

The distribution and prevalence of various species of intestinal parasites differs from region to region because of several environmental, social and geographical factors. Hence, study on the prevalence of various intestinal parasitic infections is a prerequisite not only for formulation of appropriate control strategies but also to predict risk for communities under consideration. Although several studies have been conducted on the distribution and prevalence of intestinal parasites in Ethiopia [10-12], there are still several localities for which epidemiological information is not available.

As a result of this, sanitation is likely to be particularly effective in controlling worm infections. Adults often think of sanitation in adult terms, but the safe disposal of children's faeces is of critical importance. As strategies, sanitation facilities interrupt the transmission of much faecal–oral disease at its most important source by preventing human faecal contamination of water and soil. Epidemiological evidence suggests that sanitation is at least as effective in preventing disease as improved water supply. Often, however, it involves major behavioral changes and significant household cost [13]. Therefore, the objective of the present study was to assess the prevalence of intestinal parasitic infection and associated risk factors such as environmental factors, sanitation and hygienic practices.

## Methods

### Study area

This cross-sectional study was conducted in four primary schools (Chencha Full primary school, Chemeto, Chefe, and Tolola) located at Chencha town. The town is located at an altitude of 2732 meters above sea level (masl) with 60° 15' N 37° 34' E and at about 485Km from Addis Ababa, Ethiopia. It is situated in Gamo Gofa Zone, 40 km from the nearby capital town of the zone named: Arbaminch. The area is predominately rural and most residents live in villages as agriculturists growing apple, maize, teff and other cereals.

### Sample size and sampling technique

Four hundred twenty two (422) school children were chosen to participate in the study and the sample size was determined by simple proportion formula using 40% prevalence in Chencha town [14] with a margin of error 0.05 and confidence level 95%. To minimize errors arising from the likelihood of non compliance, 10% was added to the normal sample. Number of students allocated in each school was based on the total number of students in each school divided by total number of students in the primary school. The results were multiplied by the calculated sample size. Then, students were allocated for each grade and each class room according to their educational level (Grade 1 to grade 8). Finally, the sample children were selected using systematic random sampling techniques by using class rosters as the sample frame.

### Data collection procedure and specimen examination

Interviewers were trained to conduct the survey using a pre-tested standardized questionnaire about factors such as socio demographic, environmental and behavioral factors. To ensure reliable information, the household head were interviewed in their local language.

After checking the completion of the questionnaires, a dry, clean, leak proof container labeled with the name and applicator stick were given by the technician. Proper stool samples were taken in the early morning from all selected students at their school. As soon as the stool samples were presented, all specimens were checked for their label, quantity, time, procedure of collection by a field worker (staff trained in proper hygienic and bio-safety measures) and then transported to Chencha hospital laboratory for gross and microscopic stool examinations.

In the laboratory, slides were prepared directly for wet mount in saline as well as in iodine and then were microscopically examined initially under low power (10×) bright field then under high power (40×) bright field. Simultaneously, samples were emulsified in a 10% formalin solution and transported to Arbaminch College of Health Sciences (AMCHS) Medical laboratory. From the emulsified sample 1 g (pea size) of feces was taken in about 4 ml of 10% formal water and then mixed and sieved in another tube. Then 3-4 ml of ether was added and centrifuged immediately at 750-1000 g (~ 3000 rpm) for 1 min. Finally the supernatant was discarded, and then small portion of the sediment was transferred to a slide and covered with cover slip and examined first with 10X and then 40 X objectives and again the iodine stained slides were prepared and examined microscopically.

### Quality control

Questionnaires were evaluated by collecting data from households to keep its standard in small numbers. The data consistency was checked at the site. All laboratory materials such as quality of reagents, sampling equipments, transporting system and microscope were checked in Chencha hospital by experienced laboratory professionals. The specimens were also checked for serial number, quality and procedures of collection. Concentration method was performed in AMCHS, Medical laboratory department. To eliminate observer bias, each stool sample was examined by two laboratory technicians in Chencha hospital and similar samples concentration method was performed by the college laboratory technician. In presence of inconsistent results, the results were checked by the senior and it was taken as the final result of the examination.

### Data analysis

The data were analyzed using SPSS version 16.0. During data collection completed questionnaires were checked regularly to rectify any discrepancy, logical errors or missing values. To describe data, mean and standard deviation for continuous variables and proportion for categorical variables were computed. The level of statistical significance was set as p < 0.05 and for each statistically significant factor, an odds ratio and 95% confidence interval (CI) were computed by the regression analysis.

### Ethical consideration

The college research review committee revised the proposal according to the rule and regulation. Accordingly, the proposal was approved by the Ethics Committee of Regional health bureau research and technology transfer support process. Gamo Gofa zone health department and the school administrative authorities at woreda level were informed about the study and permission was obtained. Participation was fully voluntary and informed written consent was obtained from school directors and guardians of the selected children.

## Results

### Parasitological profiles

A total of 400 school children from four schools were included in the study. Among the study subjects, 191(47.8%) were males and 209 (52.2%) were females. The mean age of study subjects was 10 years with a minimum and maximum age of 5 and 15 years respectively. The overall prevalence of Intestinal Parasitic Infections (IPIs) was 324(81.0%). Among them; 171 (42.7%) were females and 153(38.3%) were males. Higher prevalence of IPIs was found in females compared with male children. Regarding to parasitic infection in specific age group, children with age group 12–15 was more infected than 9–11 and 5–8 age groups of study subjects (85.5% vs. 80.1 and 78.1%) ,respectively. But, the difference was not statistically significant (P > 0.05) in the presence of IPIs and sex and also age. In 76 (19%) school children IPIs were not found (Table 1).

**Table 1:** Prevalence of IPIs by sex and age groups of children in Chencha primary schools, Southern Ethiopia, 2012

**Characteristics**	**Total no (%)**	**Intestinal parasites (IPs)**	
		**Positive (%)**	**Negative (%)**
**Sex**			
**Male**	191 (47.8)	154 (80.6)	37 (19.4)
**Female**	209 (52.2)	170 (81.4)	39 (18.6)
**Age groups (years )**			
**5-8**	110 (27.5)	87 (79.1)	23 (10.9)
**9-11**	186 (46.5)	148 (79.6)	38 (20.4)
**12-15**	104(26)	89 (85.6)	20 (14.4)
**Total**	400	324 (81.0)	76 (18)

Seven intestinal parasite species such as A*. lumbricoides, T. trichiura, Hookworm* species, *Hymenolopis nana, Taenia* species, *E.histolytica/dispar* and *Giardia lamblia* were identified among the primary schools children. The overall prevalence of helminthic infections was (63.8%). Among the helminthic infections, *A. lumbricoides (60.5%)* being the most predominant, followed by *T. trichiura* (9.7%), *Hookworm* species (2.2%), while only 0.8% had *Hymenolopis nana* and *Taenia* infections in primary schools. Most of helminth parasites such as *Trichuris trichuria*, *Hookwork* and *Taenia* species were higher (61.5%), (71.5%) and (65.7%), respectively in female but there was no statistically significant difference when compared with male school children (p > 0.05). With regard to protozoal infection, the overall prevalence was (23.5%). The highest prevalence rate was due to *E.histolytica/dispar* 16.2% and followed by 11.7% *Giardia lamblia.* The prevalence rate in *E.histolytica/dispar and Giardia lamblia* parasite was higher in females. However, it was not with statistically significant difference (p > 0.05) (Table 2).

**Table 2:** Distribution of IPIs to sex in Chencha town primary school children, Southern Ethiopia, 2012

**Types of parasites**	**Males**	**Females**	**Both sex**	**p-value**
	**No (%)**	**No (%)**	**No (%)**	
**Protozoans**				
*E.histolytica/dispar*	32 (49.3%)	33 (50.7%)	65 (16.25%)	P > 0.05
*Giardia lamblia*	22 (46.8%)	25 (53.2%)	47 (11.75%)	P > 0.05
**Helminths**				
*Ascaris lumbricoides*	117 (48.3%)	125 (41.7%)	242 (60.5%)	P > 0.05
*Trichuris trichiura*	15 (38.4%)	24 (61.5%)	39 (9.7%)	P > 0.05
*Taenia* species	1 (33.3%)	2 (65.7%)	3 (0.75%)	P > 0.05
Hookworm species	2 (28.5%)	7 (71.5%)	9 (2.25%)	P > 0.05
*Hymenolepis nana*	3 (100.0%)	0 (0.0%)	3 (0.75%)	P > 0.05
**Total**	153 (47.3%)	171 (52.7%)	324 (81.0%)	P > 0.05

(P < 0.05) indicates statistically significant difference.

The prevalence of helimnthic infections was observed in statistically significant difference among primary schools (P = 0.023). The prevalence was higher in Tolola primary schools (73.8%). *Ascaris lumbricoides* infections was also observed in the highest prevalence (72.5%) in Tolola with statistically significant difference (p = 0.043), followed by 22.5% *E.histolytica*/coli. *Ascaris lumbricoides* parasite was found in high prevalence rate in Mulu primary school (63.0%). Similarly compared with others, Mulu primary school children had high parasite prevalence rate (7.0%) in *Hook worm* infection. *E.histolytica*/coli and *Trichuris trichuria* were the highest prevalent parasitic infection in Chemeto primary school with (20.0%) and (15.8%), respectively. There was no statistically significant difference among the presence of IPS and primary school children except *Hookworm* and *Ascaris* infection (Table 3).

**Table 3:** Distribution of intestinal parasitic infections among Chencha primary schools, Southern Ethiopia, 2012

**Types of Parasites**	**Mulu**		**Tolola**		**Chefe**		**Chemeto**		**p-value**
	**No.**	**%**	**No.**	**%**	**No.**	**%**	**No.**	**%**	
**Total positive**	86	86.0	69	86.25	73	73.0	96	80.0	
**Protozoans**									p > 0.05
*E.histolytica/dispar*	14	14.0	18	22.5	9	9.0	24	20.0	P = 0.05
*Giardia lamblia*	12	12.0	9	11.25	18	18.0	8	6.7	
**Helminths**									P < 0.05
*Ascaris lumbricoides*	63	63.0	58	72.5	53	53.0	68	56.7	P < 0.05
*Trichuris trichiura*	12	12.0	12	15.0	5	5.0	19	15.8	
*Hook worm* species	7	7.0	0	0.0	`1	1.0	1	0.8	P < 0.05
*Taenia* species	2	3.0	0	0.0	0	0.0	1	0.8	
*Hymenolopis nana*	2	2.0	0	0.0	1	1.0	0	0.0	
None	11	16.0	24	30.0	27	27.0	14	11.7	
Single infection	63	63.0	44	55.0	60	60.0	71	59.1	
Double infection	21	21.0	19	23.7	12	12.0	24	20.0	
Multiple infection	2	2.0	6	7.5	1	1.0	1	0.8	

(P < 0.05) indicates statistically significant difference.

In this study, single infection (59.5%) was most prevalent, followed by double infections (19.0%) and multiple infections (2.5%). The combination of *Ascaris lumbricoides* and *Trichuris trichuria* were the most predominant in double infection, accounting for (9.75%) of the total study subjects. Double and multiple infections were highly prevalent in Tolola primary school children (Table 3).

### Household profiles

The risk factors associated with intestinal parasite in relation to socio-demographic character were examined by regression analysis. In this study, (56.2%) household heads were mothers/females and the remaining (43.8%) were fathers/males. The highest percentages of household heads were housewives (40.5%), (19.5%) household heads were government workers and the remaining percentage household heads were shared by merchants (14.5%), farmers (12.5) and others. Of these, (30.5%) of household heads were unable to read and write, (19.8%) were able to only read and write, (38.8%) were in primary to secondary level and (11.8%) had higher institution certificate/diploma. 80.5% of household don’t have washing facilities and only 19.5% had shower in their compound with the available material. Almost 7.8% houses were in very good cleanness condition, 80.8% houses cleanness was good and 11.5% were in bad condition.

Regarding toilet, 91.5% of the households had their own private toilet, 2.75% had a common toilet and 5.75% had no toilet. 91.5% of the household toilets were constructed in and around the compound, 8.5% was found far from their own home or in open environment. 67.5% of latrines were traditional type, 26.8% of latrines were with good air flow and 5.8% in open defecation system. Among the latrines, only 29.2% were properly constructed with good available materials. Of households who had toilets, 39.5% were with hand washing facilities and 60.5% were lacking hand washing facilities.

(75.5%) households had a pipe water source, (18.5%) had bono water source and the remaining was from the open stream. (81%) households had water deficiency problem, 10% of the households had water source interruption, (4.5%) had faced exhaustive system to collect the water, (4.0%) water sources were found in far distance and (2.0%) of individuals don’t have money to get the water from their nearest water source. (45.0%) household heads had the habits of using soap/detergents regularly, (48.0%) used soaps/detergents sometimes and (6.8%) don’t use soaps/detergents for personal hygiene purpose (Table 4).

**Table 4:** Potential risk factors associated with intestinal parasitic infections (IPIs) in Chencha, Southern Ethiopia, 2012

**Risk factors**	**No**	**IPS**		**OR (95 CI)**
		**Pos.**	**Neg.**	
Gender				
Male	191	154 (80.6)	37 (19.4)	1
Female	209	170 (81.3)	39 (18.7)	1.047 (0.635-1.726)
Education				
Unable to read and write	122	104 (85.2)	18 (14.8)	2.982 (1.362-6.53)*
only read and/write	79	70 (88.6)	9 (11.4)	4.014 (1.6-10.069)*
Primary to secondary	152	119 (78.3)	33 (21.7)	1.861 (0.91-3.89)
Higher education	47	31 (66.0)	16 (34.0)	1
Occupation				
House wife	162	137 (84.6)	25 (15.4)	1.289 (0.4-4.153)
Merchant	58	47 (81.0)	11 (19.0)	1.005 (0.282-3.586)
Government worker	79	57 (72.2)	22 (27.8)	0.61 (0.185-2.014)
Daily laborer	30	22 (73.3)	8 (26.7)	0.64 (0.167-2.513)
Farmer	50	44 (88.0)	6 (22.0)	1.725 (0.433-6.882)
Others	21	17 (81.0)	4 (19.0)	1
Home with washing facility				
Yes	78	28 (35.9)	50 (64.1)	1
No	322	296 (91.9)	26 (19.0)	20.00 (11.00-37.4997)*
Home cleanness				
Very good	31	13 (41.9)	18 (58.1)	1
Good	323	267 (82.7)	56 (17.3)	6.602 (3.059-14.249)*
Bad	46	44(95.7)	2 (4.3)	30.462 (6.234-148.852)*
Toilet ownership				
Private	366	297 (81.1)	69 (18.9)	1
Common	11	10 (90.9)	1 (9.1)	2.323 (0.293-18.453)
No	23	17 (73.9)	6 (26.1)	0.658 (0.25-1.731)
Type of latrine				
Good air flow	107	61 (57.0)	46 (43.0)	1
Traditional type	270	246 (91.1)	24 (8.9)	0.468 (0.171-1.28)
Open system	23	17 (73.9)	6 (26.1)	3.618 (1.304-10.04)*
Toilet hand washing setup				
Yes	158	124 (78.5)	(21.5)	1
No	242	200 (82.6)	42 (17.4)	1.306 (0.788-2.163)
Presence of toilet				
Close/around to home	375	299 (79.7)	76 (20.3)	1
Not in vicinity	25	25 (100.0)	0 (0.0)	4.055 (0.950-17.306)
Water supply				
Pipe water	302	234 (77.5)	62 (20.9)	1
Bono water	74	69 (93.2)	5 (6.8)	0.492 (0.142-1.698)
Stream	24	21 (87.5)	3 (12.5)	1.971(0.434-8.946)
Hand washing habit with detergent				
Yes regularly	180	141 (78.3)	39 (21.7)	1
Yes, Sometimes	193	157 (81.3)	36 (18.7)	1.206 (0.727-2.003)
No	27	26 (96.3)	1(3.7)	7.191 (0.946-54.676)

*Significant odds ratio.

### Association of risk factors with intestinal parasite infection

In this study significant relationships were observed between intestinal parasitic infection (dependent variables) and in some of socio-demographic, and environmental or behavioral factors (independent variables). Of those positive for intestinal parasites, 28 (35.9%) had washing facilities and 296(91.9%) had no washing facilities at their home with statistically significant difference (p < 0.001). Household heads who were unable to read and write and also only those who read and write had the risk of their children to acquire the intestinal parasitic infection than household heads who had higher educational level with statistically significant difference (p < 0.05). In addition, bivariate analysis showed no significant association between the prevalence of intestinal parasite infection and the occupation of their parents (Table 4).

Of those who had intestinal parasite 44(95.7%) had poor cleanness condition than households who had a very good cleanness condition 13 (41.9). This showed that households with poor cleanness condition had a more likelihood to be infected than those who had very good cleanness condition (Odds ratio 30.42; 95% CI 6.234 to 148.852, p < 0.01). Households who used a common toilet had the risk to be infected by intestinal parasite infection than those who had private toilet (Odds ratio 2.323; 95%CI 0.293 to 18.453). However, the association was not statistically significant (p > 0.05). In addition, there was no statistically significant difference in the prevalence of IPIs between those who had toilets with hand washings facilities in the households and those who had no the facilities (p > 0.05) (Table 4).

School children who used an open defecation system were the more to be infected than the other type of latrine with statistically significant difference (Odds ratio 3.618; 95% CI 1.304 to 10.04, p < 0.05). Likewise, school children who used traditional type of toilet had the highest prevalence of IPIs. In addition, households who had the toilet far from vicinity were the more likelihood to be infected with IPIs than households who had the toilet at their home or around. However, the difference was not statistically significant (Odds ratio 4.055; 95% CI 0.95 to 17.306). Using pipe water source was more protective from IPIs than those who used stream and bono water source (0.34; 95% CI 0.13 to 0.74). Household heads who had no habits of washing their hands with soap/ detergent had the more likelihood their children to acquire the parasitic infection than those who had the habit of washing their hands with soap/detergents regularly without statistically significant association (odds ratio 7.191; 95% CI 0.946 to 54.676) (Table 4).

## Discussions

It is known that the transmission of intestinal parasites depends on the presence of infected individuals, poor sanitation and principally, the socioeconomic and behavioral factors in the population. This study attempted to show the potential risk factors for the prevalence of intestinal parasitic infection in primary school children. The overall prevalence of intestinal parasite was (81.0%) and it was consistent with the study which was conducted at Delgi primary school, Northern Ethiopia [15]. However, this figure is very high compared with other similar studies in Ethiopia [11-13]. Similarly, high prevalence has been consistently reported by in a number of countries in school children [16,17].

Age is important risk factor for the prevalence of intestinal parasitic infection. In this study relatively highly prevalent parasitic infection was observed in high age range of children. This could be due to the fact that as the child grows older the exposure to different risk factors for intestinal parasite infection increases. In contrast, other study showed that the prevalence was found to be significantly high in children with lower age [2]. This might be due to older age children are comparatively more knowledgeable and aware than the lower age children to be infected with intestinal parasite.

Results also showed that STH infections (63.8%) were more common compared with protozoan infections (23.5%). *Ascaris lumbricoides* accounted the highest prevalence of intestinal parasite (60.5%). This finding showed higher *Ascaris* infection than the previous studies which were conducted in Ethiopia where *Ascaris lumbricoides* was the highest prevalent [11,15,18] and other countries [19,20]. In agreement with the global data, *Ascaris lumbricoides* infections were among the three STH in the current study. The hot and humid weather and wet soil favoured for the presence of the parasite in this study area.

On the other hand, other intestinal helminthic infection prevalence rate varies in the schools. *T.trichuria* was found to be the second most prevalent (9.7%) soil transmitted helminthes in this study. This prevalence was lower compared with other study in Ethiopia [15] and other country [2]. *Hook worm, Taeina species* and *Hymenolopis nana* infection rate were found to be lower than the previous studies [11,15].

As for protozoal infection, the overall prevalence was (23.5%) lower than other study. In agreement with other studies in Ethiopia the prevalence of *E.histolytica* (16.2%) was very high [12,16]. But, this was not consistent with the study which was conducted in North Gondar, Ethiopia [15]. In contrast, in the current study the prevalence of *G.lamblia* (11.7%) was higher compared with other study in Ethiopia [12].

In our study, single, double and multiple infections (59.5%, 19.0% and 2.5%) were observed respectively. Single infection had higher prevalence rate than other studies [12,15]. Similarly, most of double infections were by *Ascaris* and *Trichuris* infection [21].

This study assessed the association of potential risk factors with the prevalence of intestinal parasitosis among school children. Socio-demographic, Environmental, behavioral factors and different sanitation facilities had a significant contribution for the presence of IPIs [22]. One of the factors with strongly statistically significant difference was the presence of intestinal parasitic infection in parents/guardians of school children who had low levels of education (OR 2.982; 95% CI 1.362 to 6.53 and OR 4.014; 95% of CI 1.6-10 to 069, p < 0.05). This finding was consistent with other study in school [23].

Among the potential risk factors, the unavailability of washing facilities constructed at home had also a contributing effect for the presence of intestinal parasitosis. Home cleanness condition also had contribution for the existence of IPIs. In this study, 94% of school children had an access to safe drinking water from either of the pipe or Bono water sources and 94.25% of them also had an access to latrine facilities. The facilities could have some contribution to lower prevalence rate of intestinal protozoa infections in Chencha. In contrast, Delgi School children in south Gondar of Ethiopia [15] only 56% children had an access to safe drinking water and 17.3% had latrine facilities with lower STH compared to Chencha school children. Conversely, the availabilities of the above facilities at their home might have no contribution to reduce the prevalence rate of STHs in our study. The findings indicated that the source of infection might be due to improper use of facilities in households, and the unavailability of the facilities at the schools.

The finding also showed school children who had no toilet with washing facilities in their home were more likely to acquire the IPIs than those who had the facilities. However, the difference was not statistically significant (p > 0.05). An open defecation system of latrine in the living environment could have a significant contribution for the occurrences of IPIs (P < 0.05). The highest prevalence of IPIs was also found in children who had no toilet at their vicinity compared to those who had toilet at/around their home. This might have contribution due to the absence washing facility and exposure of children to parasites in open defecation system.

The present study found out that household heads that had no washing habits were more likely to be infected with intestinal infection. This reason may show household heads especially mothers who don’t have the habit of washing their hands regularly contaminate the food while they were involved in the preparation. However, further investigation is needed to find other unknown factors which may contribute as potential risk factors. As a limitation of this study, identification of Cryptosporidium cases and the pathogenic *E.histolytica* from the enteric commensals *E.dispar* were not differentiated due to lack of laboratory facilities in the department. Although the potential risk factors for high burden of the disease were considered, some risk factors were not evaluated in the current study. Apart from this, it is a good reference study on risk factors leading to intestinal parasitosis. It is also the first study which indicates the consequence of social, local factors related to home and school duties, parents’ behaviors and hygienic condition for the burden of disease in this study area.

## Conclusions

The prevalence of intestinal parasitic infections, especially soil-transmitted helminths (STHs) is very high in the school children. The high prevalence of parasitic infections in these populations of children indicates that the protozoa and helminthes concerned are very common in the environment of these villages and the results of the risk factors analysis suggests that transmission is from several routes. Therefore, multiple intervention strategies should be implemented for the school children, households and environment for reduction of intestinal parasitic infections.

## Competing interests

The author(s) declare that they have no competing interests.

## Authors’ contributions

AA Conception of the research idea, designing, collection of data, data analysis, interpretation, and manuscript drafting. MS Designing, collection of data and manuscript drafting. All authors read and approved the final manuscript.

## Pre-publication history

The pre-publication history for this paper can be accessed here:

http://www.biomedcentral.com/1471-2458/14/166/prepub
